# Correction: ‘Unwilling’ *versus* ‘unable’: Tonkean macaques’ understanding of human goal-directed actions

**DOI:** 10.7717/peerj.3227/correction-1

**Published:** 2019-11-07

**Authors:** Charlotte Canteloup, Hélène Meunier

**Affiliations:** 1 Centre de Primatologie de l’Université de Strasbourg, Niederhausbergen, France; 2 Université de Strasbourg—Laboratoire de Neurosciences Cognitives et Adaptatives, Strasbourg, France; 3 CNRS—Laboratoire de Neurosciences Cognitives et Adaptatives, UMR 7364, Strasbourg, France; 4 University of Strasbourg Institute for Advanced Study, Strasbourg, France; 5 Anthropological Institute and Museum, University of Zürich, Zürich, Switzerland

Corrigendum: 

After publication, the writers were informed of errors in the statistical analysis. Accordingly, the authors have conducted new statistical analyses following the advice of statisticians. The following changes have been made to correct these issues: 

In the Abstract, the fourth sentence now reads: “They attempted to grasp food in the actor's hand significantly more in the presence of an unwilling actor rather than an unable or a distracted one.” The fifth sentence now reads: “Inversely, they begged significantly more facing a distracted and unable experimenter rather than an unwilling one.”

In the Materials and Methods, at the beginning of the Apparatus section: the authors have now specified that all the fifteen subjects were tested within their social group in an outdoor area. 

This sentence has been added to the second paragraph of the Experimental procedure section: “In total, each subject was tested six times in each of the three experimental conditions that consisted in a total of 18 experimental trials per individual.”

In the Statistical Analysis section, the third sentence now reads: “Second, because experimental trials did not last exactly the same, we fitted Linear mixed-effects models (LME) for logit transformed proportion data in order to test which experimental conditions influenced continuous variables as the proportion of time spent in the following behaviors: item grasp attempt, gaze elsewhere and yawn and self-scratch.” A new sentence has been added after the third stating that: “Normality of the residuals was assessed graphically (QQ-plots).”

In the Results, at the beginning of the Presence of the subject section, “mean proportion” has been corrected to “mean percentage”. The same change has been made in the Figure 2 legend. For the sake of clarity, percentage of time spent in the different behaviors instead of proportions are displayed in the figures.

In the second paragraph of the Gaze section, the original LRT value has been corrected to 35.352. The second sentence of this paragraph now reads: “According to LME, macaques looked elsewhere for significantly longer in the ‘distracted’ condition compared with the ‘unwilling’ condition (32.06% ± 2.37; P <0:0001), and in the ‘unable' condition compared with the ‘unwilling' condition (P <0.0001).” A new sentence has been added to the end of this section, stating that: “There were no significant differences between the ‘unable’ condition (46.79 % ± 2.04) and the ‘distracted’ condition (45.52% ± 2.41; P >0.05).”

In the second paragraph of the Gestures section, the original LRT value has been corrected to 129.93. GLMM has been replaced with LME. The statistical significance of macaques spending more time trying to grab the item in the ‘unable’ than the ‘distracted’ condition has been corrected so that is equal to 0.00066. 

The Threat, yawn, and self-scratch section has been updated to read: “Only four individuals over fifteen displayed threat behaviors towards the experimenter at a very low level in the ‘unwilling’ (0.48% ± 0.17), ‘unable’ (0.09% ± 0.09) and the ‘distracted’ conditions (0.02% ± 0.02).

The proportion of time yawning and self-scratching (Fig. 4) was significantly influenced by the experimental condition (LRT = 8.744; Df = 2; P = 0.012). LME revealed significantly more time in these behaviors in the ‘distracted’ (4.95% ± 1.01) than the ‘unwilling’ condition (2.33% ± 0.61; P = 0.015). However, there were no significant differences neither between the ‘unwilling’ and the ‘unable’ conditions (P>0.05) and between the ‘unable’ (2.78% ± 0.72; P>0.05). Macaques tended to display more yawn and self-scratch behaviors in the ‘distracted’ than ‘unable’ condition (P = 0.057). 

In the first paragraph of the Discussion, observations regarding Tonkean macaques displaying more frustration behaviors when facing an unable experimenter versus an unwilling one have been removed. 

The first sentence of the second paragraph now reads: “Tonkean macaques displayed significantly more gaze alternation between the experimenter's face and hand, and tried to grasp the item significantly more in the ‘unwilling’ than the ‘unable’ and ‘distracted’ conditions, and in the ‘unable’ than the ‘distracted’ condition.” In the thirteen sentence, macaques’ “aggressive and gestural” behaviors have corrected to “grasping attempt” behavior. 

The fourteenth sentence of the paragraph now reads: “Indeed, we reported that Tonkean macaques attempted significantly more to grasp food when the experimenter was unwilling to give them food than when she was unable or distracted to do so.”

The nineteenth sentence now reads: “On the other hand, another way to test for the frustration hypothesis would be analyze results of agonistic behaviors and frustration behaviors displayed by macaques as yawning and self-scratching (Maestripieri et al., 1992).” 

The second paragraph in the Discussion now ends with: “Subjects displayed significantly more frustration behaviors in the ‘distracted’ condition than the ‘unwilling’ one, and they tended to show more such behaviors facing a distracted experimenter rather than an unable one. This could be interpreted as a frustration of not obtaining the food visible on the table but out of reach in the ‘distracted’ condition compared to the other ones. Despite the fact that very few threats have been recorded and that no statistical analyses could then be run, it is nonetheless interesting to notice that, when threats occurred, it happened more facing an unwilling experimenter rather than an unable or distracted one. However, more data are necessary to strengthen the explanation that Tonkean macaques perceive the goals of the human actions.”

The Acknowledgements have been updated to specify that Nicolas Poulin is from Strasbourg University. Both Nicolas Poulin and Frédéric Schütz (Lausanne University) are thanked for their assistance with the statistical analysis. 

The figure 2 legend now reads: “(A) Mean number of gaze alternations between the experimenter and her hand holding the item per trial. (B) Mean percentage of time (±standard error of the mean) macaques looked elsewhere per trial.” 

The Figure 3 legend now reads: “(A) Mean number of begging gestures (±standard error of the mean) and (B) mean percentage of time of time (±standard error of the mean) macaques spent attempting to grasp the item in the experimenter's hand per trial.”

The Figure 4 title and legend now read: “Yawn and self-scratch. Mean percentage of time macaques (±standard error of the mean) spent displaying yawn and self-scratch per trial.”

Figures 2, 3, and 4 have been revised according to the statistical changes in results. In Figure 2, the significant difference previously found between the ‘unable’ and ‘distracted’ conditions has been removed. In Figures 2 and 3, the Y axis has been labelled “mean percentage of time (%)” instead of “mean proportion of time (%)”. In Figure 4, the A part corresponding to the threats has been removed. Only yawn and self-scratch are now displayed, and the significant differences previously found between ‘unwilling’ and ‘unable’ conditions and between ‘unable’ and ‘distracted’ conditions have been removed. 

Lastly, Charlotte Canteloup’s fifth affiliation now reads: “Department of Ecology and Evolution, University of Lausanne, Lausanne, Switzerland”. 

Original PDF

